# A Robust Numerical Methodology for Fatigue Damage Evolution Simulation in Composites

**DOI:** 10.3390/ma14123348

**Published:** 2021-06-17

**Authors:** Angela Russo, Andrea Sellitto, Prisco Curatolo, Valerio Acanfora, Salvatore Saputo, Aniello Riccio

**Affiliations:** Department of Engineering, University of Campania “L. Vanvitelli”, Via Roma 29, 81031 Aversa, Italy; andrea.sellitto@unicampania.it (A.S.); prisco.curatolo@studenti.unicampania.it (P.C.); valerio.acanfora@unicampania.it (V.A.); salvatore.saputo@unicampania.it (S.S.); aniello.riccio@unicampania.it (A.R.)

**Keywords:** fatigue, residual strength, residual stiffness cycle jump strategy, open-hole specimen

## Abstract

Composite materials, like metals, are subject to fatigue effects, representing one of the main causes for component collapse in carbon fiber-reinforced polymers. Indeed, when subject to low stress cyclic loading, carbon fiber-reinforced polymers exhibit gradual degradation of the mechanical properties. The numerical simulation of this phenomenon, which can strongly reduce time and costs to market, can be extremely expensive in terms of computational effort since a very high number of static analyses need to be run to take into account the real damage propagation due the fatigue effects. In this paper, a novel cycle jump strategy, named Smart Cycle strategy, is introduced in the numerical model to avoid the simulation of every single cycle and save computational resources. This cycle jump strategy can be seen as an enhancement of the empirical model proposed by Shokrieh and Lessard for the evaluation of the fatigue-induced strength and stiffness degradation. Indeed, the Smart Cycle allows quickly obtaining a preliminary assessment of the fatigue behavior of composite structures. It is based on the hypothesis that the stress redistribution, due to the fatigue-induced gradual degradation of the material properties, can be neglected until sudden fiber and/or matrix damage is verified at element/lamina level. The numerical procedure has been implemented in the commercial finite element code ANSYS MECHANICAL, by means of Ansys Parametric Design Languages (APDL). Briefly, the Smart Cycle routine is able to predict cycles where fatigue failure criteria are likely to be satisfied and to limit the numerical simulation to these cycles where a consistent damage propagation in terms of fiber and matrix breakage is expected. The proposed numerical strategy was preliminarily validated, in the frame of this research study, on 30° fiber-oriented unidirectional coupons subjected to tensile–tensile fatigue loading conditions. The numerical results were compared with literature experimental data in terms of number of cycles at failure for different percentage of the static strength. Lastly, in order to assess its potential in terms of computational time saving on more complex structures and different loading conditions, the proposed numerical approach was used to investigate the fatigue behavior of a cross-ply open-hole composite panel under tension–tension fatigue loading conditions.

## 1. Introduction

Composite materials are commonly used today in many engineering and industrial fields and, often, can be considered the first choice for structural load-bearing components [1,2,3,4]. The scientific community is increasingly interested in robust numerical procedures capable of correctly predicting the mechanical behavior of such innovative materials, especially in terms of damage propagation. Indeed, failure mechanisms are a major weakness for composites, which hinders fulfilling certification regulations [5,6] and leads to oversizing of the structures without achieving the promised improvements in terms of weight reduction.

For decades, robust numerical methods have been developed to investigate the behavior of composite materials subjected to static loading conditions [7,8,9]. In [10], the damage behavior of an aerospace stiffened panel made of epoxy resin/carbon fiber material, subjected to static compressive load, was studied experimentally and numerically. Hill [11], Tsai Wu [12], and Hashin and Rotem [13,14] can be considered pioneers in developing mathematical models describing the onset of intralaminar damage and evolution of composite materials subjected to static loading conditions. They defined the mode-dependent failure criteria which allow calculating the fiber and matrix breakages on the basis of the state of stress and the material strength. Today, such models are implemented in all the main commercial finite element platforms, and they have inspired most of the damage prediction methodologies reported in the literature.

The same level of knowledge and confidence has not been reached when cyclic loading condition are considered. The phenomenon of material property degradation caused by cyclic loads is called fatigue and, even if discovered in the second half of the 19th century for metallic materials, this term has been commonly extended to other classes of materials, including composites. Currently, the knowledge about composite response under fatigue, particularly carbon fiber-reinforced polymers (CFRPs), has made excellent progress, but the development of robust computational methods to correctly predict the fatigue life of structures is still in progress [15].

In recent years, different numerical and experimental models have been proposed to predict the behavior of composite structures under cyclic loads applied over time. These models are frequently clustered in two major groups: fatigue life models, which predict fatigue life without focusing on the property degradation mechanisms of evolution, and phenomenological models, which predict the evolution of damage and allow evaluating the residual stiffness and strength degradation over cycles.

The fatigue life models [16,17,18,19,20,21,22] make use of experimental data from constant amplitude fatigue experimental tests, considering different levels of stress, as described by Adam et al. in [23,24], where the fatigue behavior of T800/5245B composite samples was assessed in tension, compression, and mixed tension–compression, allowing a prediction of the so-called S–N curves, correlating the number of cycles to failure to the applied stress level. The principal drawback of these models is that they need massive experimental data, which require costly and time-intensive experimental campaigns.

The phenomenological models can describe the stiffness and strength degradations due to the accumulation of damage. These are based on experimental measurements at different stress levels and different fractions of their fatigue life. Empirical laws are used to fit these test data, providing evolution laws that can describe the gradual reduction in laminate stiffness and strength at a macroscopic level.

Several phenomenological models have been proposed for stiffness degradation [25,26]. For example, in [27,28,29,30], some models were developed to predict the stiffness reduction which characterizes the types of damage that commonly arise during fatigue, as well as the strength degradation [31,32] under fatigue loading conditions. Shokrieh et al. [33,34,35,36,37,38] proposed an empirical method based on the reduction in strength and stiffness of the material, called the generalized residual material property degradation model, which establishes a technique taking into account the fatigue-driven damage caused by arbitrary stress ratio, without the need for excessive amounts of testing. More in detail, this approach integrates the residual strength and stiffness theory with the Hashin fatigue failure criteria, for different damage mechanisms. The model, named the “residual strength material properties degradation model”, has been extensively employed by many authors to implement material user subroutines for use within proprietary and commercial finite element codes. Naderi and Maligno in [39] proposed a three-dimensional Finite Elements (FE) model to simulate the fatigue response of AS4/3501-6 carbon/epoxy samples. They implemented the progressive fatigue model in ABAQUS™ via user subroutines UMAT (user-defined material) and USDFLD (user-defined field variables). Similarly, Krishnan, Conway, and Xiao in [40] presented a material user defined routine for ABAQUS™ used to simulate fatigue behavior of specimens with a central circular hole. They performed experimental tests under tensile fatigue loading conditions and monitored the strains and displacements by a digital image correlation device to validate their numerical models. Khan et al. in [41] also developed a user material subroutine (UMAT) based on the Shokrieh and Lessard model. They used a cumulative damage approach to assess the variation in stress amplitude, resulting from the stress state redistribution after failure.

In this work, the Shokrieh and Lessard fatigue empirical model was implemented in the commercial Finite Elements Method (FEM) software ANSYS^®^ by means of the Ansys Parametrical Design Language (APDL) with the aim of developing a robust, easy-to-use, and fast numerical procedure able to preliminary assess the fatigue life of composite structural components. The main goal of this paper was to validate a cycle jump strategy, labeled the Smart Cycle strategy, able to predict the cycles where fatigue failure criteria are likely to be fulfilled, assuming that the stress redistribution, due to the fatigue gradual degradation of the material properties, can be neglected until sudden fiber and/or matrix damage is verified at the element/lamina level. Hence, the Smart Cycle strategy is able to limit the cycles where numerical simulations are needed, allowing to save computational costs and storage use. The implemented finite element procedure was preliminary validated against experimental data available in the literature for three different samples and applied to open-hole specimens under tensile fatigue conditions.

In Section 2, the theory behind the implemented model is introduced, while, in Section 3, the finite element model and the Smart Cycle strategy implementation are presented. In Section 4, the developed numerical tool is preliminarily validated against experimental data on an off-axis unidirectional specimen subjected to tensile–tensile fatigue and applied to cross-ply open-hole specimens under constant-amplitude tensile fatigue. The comparison of the obtained numerical results to the literature experimental measurements demonstrates the effectiveness of the Smart Cycle strategy in saving computational costs without loss of results accuracy.

## 2. Theoretical Background

**Figure 1:**
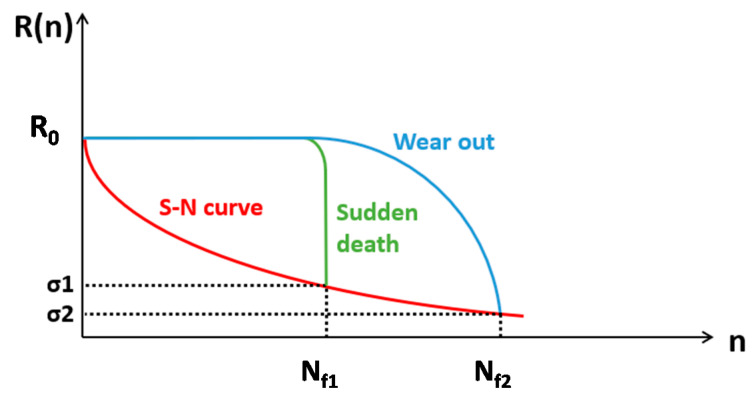
Schematic representation of the strength degradation under variable stress conditions.

As already remarked, the fatigue intralaminar damage evolution approach proposed in this paper is based on the Shokrieh and Lessard’s residual strength material property degradation model [34,35]. In Figure 1, the residual strength and S–N curves, referring to a unidirectional lamina, are shown in one graph. For each state of stress, the S–N curve intersects the catastrophic failure point of the residual strength curve. Let us consider, in the schematic curve of Figure 1, the static strength of a unidirectional lamina *R*_0_, which can also be expressed as the strength at one-quarter of a cycle (*n* = 0.25) in fatigue where the maximum value of the applied stress is reached. Under a constant maximum applied stress (*σ*), the fatigue strength R(n) decreases until it reaches the magnitude of the maximum applied stress. At this point, R(N_f_) in Figure 1, the lamina fails catastrophically.

The fatigue behavior of a composite lamina depends on the applied stress. Two different models can be identified: the sudden death model and the wear-out model.

According to Figure 1, when subjected to a high level of stress, the residual strength as a function of the cycle number is almost constant until it decreases abruptly (sudden death model). On the contrary, under a low level of stress, the residual strength degrades gradually (wear-out model). The Shokrieh and Lessard model is capable of capturing both the “sudden death” and “wear-out” models for failure in laminates.

### 2.1. Wear-Out Model

To fully describe the generalized residual material property degradation model, three main expressions must be combined: the normalized residual strength, the normalized residual stiffness, and the normalized fatigue life model.

Referring to the residual strength of a unidirectional lamina under general uniaxial fatigue loading conditions, a suitable relationship among fatigue life, state of stress, and stress ratio is needed. The expression, proposed by Harris et al. [22,23,24], was rearranged, as shown in Equation (1), by Shokrieh and Lessard in [34,35].

(1) $$R\left(n,\sigma ,k\right)={\left[1-{\left(\frac{log\left(n\right)-log\left(0.25\right)}{log\left(N_{f}\right)-log\left(0.25\right)}\right)}^{\beta }\right]}^{\frac{1}{\alpha }}\left(R_{0}-\sigma \right)+\sigma$$

Knowing the static residual strength *R*_0_, the state of stress *σ*, and the experimentally determined curve-fitting parameters *α* and *β*, the residual strength as a function of the number of cycles *n* and stress state *σ*, for arbitrary stress ratio *k*, can be determined. In addition to the normalized residual strength model, the normalized residual stiffness *E*(*n*,*σ*,*k*) model is described in Equation (2), providing the residual stiffness of a unidirectional ply under a certain state of stress σ and stress ratio *k*.

(2) $$E\left(n,\sigma ,k\right)={\left[1-{\left(\frac{log\left(n\right)-log\left(0.25\right)}{log\left(N_{f}\right)-log\left(0.25\right)}\right)}^{\lambda }\right]}^{\frac{1}{\gamma }}\left(E_{s}-\frac{\sigma }{\varepsilon_{f}}\right)+\frac{\sigma }{\varepsilon_{f}}.$$

According to Equation (2), *Es* is the static stiffness, and *γ*, *λ*, and *ε_f_* (average strain to failure) are additional experimental fitting parameters. Even if the experimental fitting parameters shown in Equations (1) and (2) are stress-independent, the number of cycles to failure (*N_f_*) is a function of the state of stress and the stress ratio.

**Figure 2:**
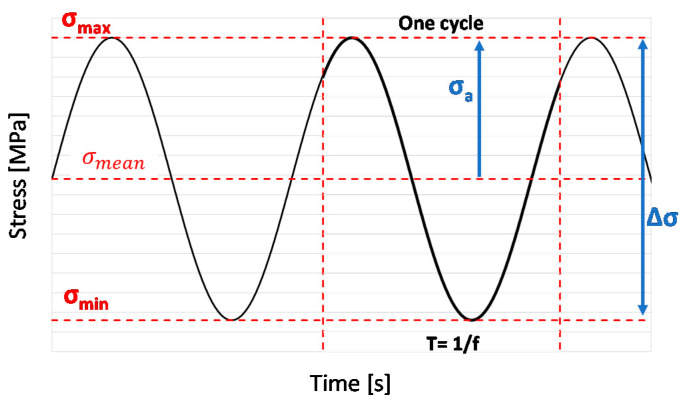
Constant-amplitude loading pattern.

Lastly, the normalized fatigue life model as a function of the cycles to failure *N_f_*, at lamina level, is evaluated using the expression in Equation (3), developed by Adam et al. [23].

(3) $$u=\frac{ln\left(a/f\right)}{ln\left[\left(1-m\right)\left(c+m\right)\right]}=A+Blog\;N_{f},$$

where $m=\frac{\sigma_{mean}}{\sigma_{t}}$, $c=\frac{\sigma_{c}}{\sigma_{t}}$, and $a=\frac{\sigma_{alt}}{\sigma_{t}}$, with *σ_t_* tensile stress, *σ_c_* compressive stress, $\sigma_{mean}=\frac{\left(\sigma_{max}+\sigma_{min}\right)}{2}$ mean stress, and $\sigma_{a}=\frac{\left(\sigma_{max}-\sigma_{min}\right)}{2}$ alternating stress shown in Figure 2, where a constant-amplitude loading pattern is represented. The terms *f*, *A*, and *B* are curve-fitting parameters which can be experimentally determined as described in [35].

Equations (1)–(3) can be written for each stress direction (longitudinal, transverse, and shear directions) and, where applicable, for both tensile and compressive loading conditions, considering the proper stress components, number of cycles to failure, static strength, maximum stress components, static stiffness, and experimental parameters *f*, *A*, *B*, *α*, *β*, *γ*, *λ*, and *ε_f_*.

### 2.2. Sudden Death Model

In addition to the gradual degradation of the material properties, described in the previous paragraph, sudden degradation, occurring as a consequence of failures at lamina level for a specific location, has to be considered to assess the damage mechanisms developed under fatigue loading conditions.

In order to check for the occurrence of failures, the fatigue failure criteria proposed by Hashin [13,14] were chosen as reported in Table 1. In the sudden degradation model, all the properties are reduced one time, instantaneously, to a fraction of the undamaged properties.

According to Table 1, the denominators of the equations are not constants but functions of the number of cycles, the state of stress, and the stress ratio. By using the expression in Equation (1) to define the residual strength for each state of stress (*σ*_11_, *σ*_12_, *σ*_13_, etc.), the expression in Equation (4) can be obtained, for example, for the matrix tensile failure.

(4) $${\left(\frac{\sigma_{22}}{{\left[1-{\left(\frac{log\left(n\right)-log\left(0.25\right)}{log\left(N_{f_{22}}\right)-log\left(0.25\right)}\right)}^{\beta_{22}}\right]}^{\frac{1}{\alpha_{22}}}\left(Y_{T}-\sigma_{22}\right)+\sigma_{22}}\right)}^{2}\;+{\left(\frac{\sigma_{12}}{{\left[1-{\left(\frac{log\left(n\right)-log\left(0.25\right)}{log\left(N_{f_{12}}\right)-log\left(0.25\right)}\right)}^{\beta_{12}}\right]}^{\frac{1}{\alpha_{12}}}\left(S_{XY}-\sigma_{12}\right)+\sigma_{12}}\right)}^{2}=1,$$

where $\alpha_{22}$ and $\beta_{22}$ are experimental parameters measured from transverse tensile fatigue tests, and $\alpha_{12}$ and $\beta_{12}$ are experimental parameters measured from the in-plane shear fatigue tests.

When failure occurs, a degradation factor $\bar{k}$ is considered to degrade the appropriate material property. Actually, properties are not reduced to 0 in order to avoid ill-conditioning of the stiffness matrix and convergence problems. However, sensitivity analysis can be performed to select the degradation factor.

## 3. Finite Element Model and Smart Cycle Strategy Implementation

**Figure 3:**
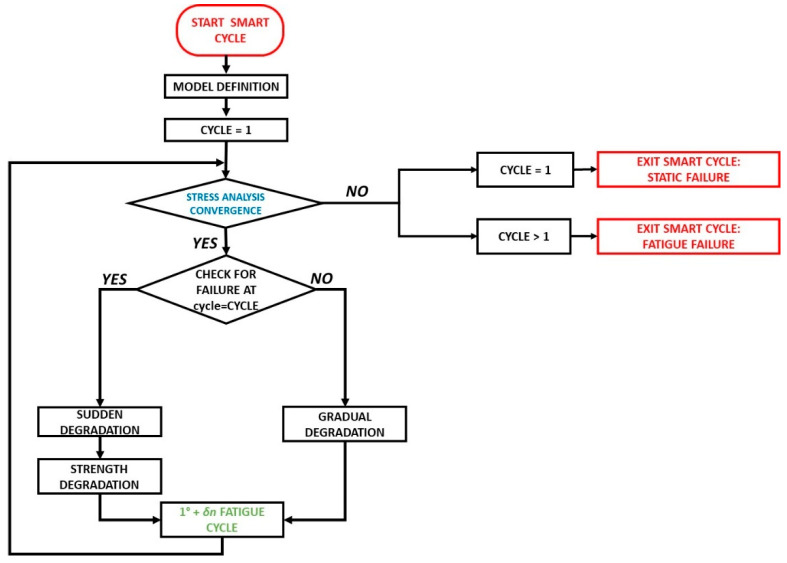
Flowchart of the numerical procedure.

The residual strength material property degradation model was implemented in the ANSYS^®^ Finite Elements software (v18.0, 2018, Ansys, Inc., Canonsburg, PA, USA) by means of the Ansys Parametric Design Language (APDL). A flowchart schematically representing the FEM implementation is presented in Figure 3. According to Figure 3, as a first step, the finite element model is defined (geometry, material proprieties, boundary conditions, minimum and maximum fatigue loads, maximum number of cycles, and cycle increments). Then, a first static analysis, under displacement mode control, can be performed to assess the ultimate static failure load, if the value has not been obtained experimentally, by determining the number of cycles to failure using Equation (3) The stress analysis is subsequently performed, by applying the proper load, cycle by cycle. Once the convergence is achieved within a cycle, the next cycle is selected considering a predefined cycle increment δn (which can be balanced considering the load percentage and the number of cycles to failure), and the proper gradual material degradation rules are applied to all the elements at lamina level. If damage is detected within an element, the mechanical properties are instantaneously degraded, according to the sudden death model, considering a degradation factor $\bar{k}=0.1$. Successively new stress analysis is performed with the degraded material properties until the maximum cycle number or the number of cycles to failure is reached. All the information about the fatigue life, the damage, and the residual material properties, for all the elements of each lamina, is stored in a database for every cycle and load step.

With the aim of avoiding the simulation of every single cycle and saving computational resources, a cycle jump strategy, named Smart Cycle, was introduced into the numerical model. The main aim was to understand the validity of the key hypothesis of negligible effects of the stress redistribution due to the gradual degradation of material properties before sudden damage onset on the overall fatigue behavior and on the determination of the fatigue life.

The Smart Cycle routine is able to predict the cycles where fatigue failure criteria are likely to be verified. Hence, only the numerical simulations to the cycles where damage propagation (in terms of fiber and matrix breakage) is expected are carried out.

The Smart Cycle strategy assumes that the stress redistribution, due to the fatigue gradual degradation of the material properties, can be neglected until sudden fiber and/or matrix damage is verified at the element/lamina level. Hence, as a function of the first fatigue cycle stress distribution, the relationships in Table 1 are checked in each lamina of each element considering the degraded material fatigue strengths. This check is repeated for each cycle, adopting the stress of the first fatigue cycle until a sudden fiber or matrix failure is detected.

### Strategies to Reduce Computational Time

**Figure 4:**
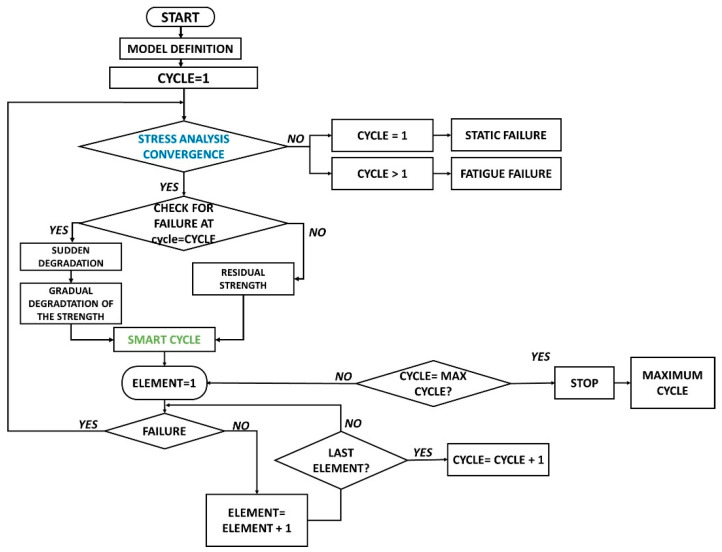
Flowchart of the numerical procedure with the introduction of the Smart Cycle strategy.

To save computational time, the matrix failure criteria are not evaluated if fiber failure is detected within an element. When a matrix or fiber failure in a lamina is verified, the mechanical properties related to the damaged elements are instantaneously degraded, while the gradual degradation of material properties is applied to all the other elements. Then, the numerical simulation is performed for the selected cycle. The same procedure is repeated starting from the cycle where sudden damage is detected, searching for the next cycle with a sudden damage onset and updating the gradual degradation just in case a sudden failure is detected, as shown in the flowchart in Figure 4.

In order to further decrease the computational cost, it is possible to do the following:

- choose the number of elements where the Hashin criteria needs to be satisfied (default setting is =1) to perform a full numerical analysis with sudden degradation in damaged elements and gradual degradation in all the other elements. This would allow reducing the number of numerical simulations to be performed by grouping the sudden damage for the selected number of elements in one fatigue cycle. This further option would, surely, save additional computational cost but would, probably, cause a decrease in accuracy with an underestimation of the damage evolution;
- choose the ΔN cycles where the check of the Hashin failure criteria is performed (default setting is =1). This would allow speeding up the smart cycle check with a decrease in the computational cost but would also decrease the accuracy in determining sudden damage onset.

Such additional computational cost-saving features would be useful when analyzing the fatigue behavior of complex structures or when very low load levels are used in fatigue cycles.

On the other hand, the accuracy of the Smart Cycle strategy can be improved (with an increase in computational costs) by performing additional stress analyses with application of gradual degradation of the material properties in all elements a few cycles (the number could be set in input, default is set = 0) before the sudden damage onset is expected according to the Hashin failure criteria check.

In this paper, the basic Smart Cycle strategy (with default values for additional parameters related to computational cost–accuracy balance) was investigated.

## 4. Smart Cycle Strategy Validation

The proposed Smart Cycle strategy was preliminary validated on 30° fiber- oriented unidirectional coupons subjected to tensile–tensile fatigue loading conditions. The numerical results were compared with the standard fatigue method (considering the predefined increment) and experimental data from the literature in terms of number of cycles at failure for different percentages of static strength. Lastly, in order to assess its potential in terms of computational time saving on more complex structures and different loading conditions, the Smart Cycle was used to investigate the fatigue behavior of a cross-ply open-hole composite panel under tension–tension fatigue loading conditions.

### 4.1. Off-Axis Tensile Specimen

The implemented ANSYS MECHANICAL^®^ APDL procedure was preliminary validated by comparing the numerical results with the literature experimental data of the off-axis unidirectional specimen subjected to tensile–tensile fatigue in [35].

**Figure 5:**
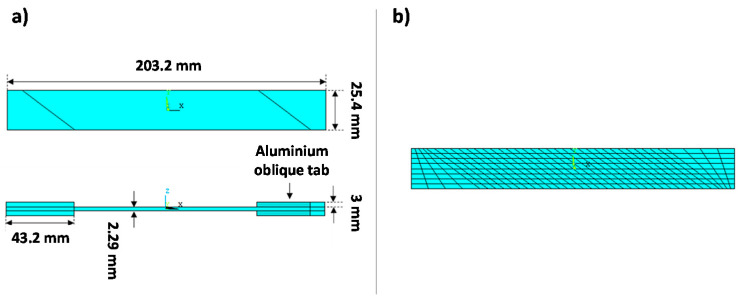
Off-axis tensile specimen: (**a**) numerical model; (**b**) Finite Elements model.

The numerical model, with the geometrical dimensions, is shown in Figure 5a. The specimen was discretized by means of four-node SHELL181 ANSYS layered elements with a reduced integration scheme. The discretization was chosen according to a previously made mesh convergence analysis which is not reported here for the sake of brevity [30]. A unidirectional AS4/3501-6 carbon fiber/epoxy matrix material system has been considered, with a ply thickness of 0.146 mm. The finite element model is shown in Figure 5b.

**Figure 6:**
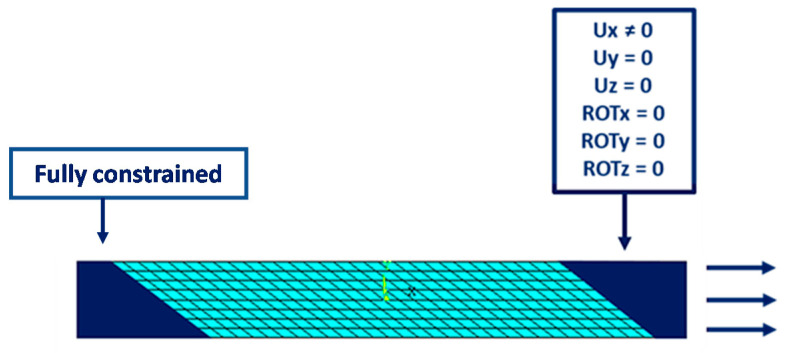
Off-axis tensile specimen: Boundary conditions.

The boundary conditions, representing the tensile–tensile fatigue load, are shown in Figure 6.

The AS4/3501-6 carbon fiber/epoxy matrix material system properties taken from [35] were considered in our numerical model. In Table 2, the mechanical properties are reported, while the experimental fitting parameters, extrapolated as described in [35], can be found in Figures 4, 5, 7, 9–11, 13, 15–17, 19, 20 and 22 of [38] for different loading directions and conditions.

**Figure 7:**
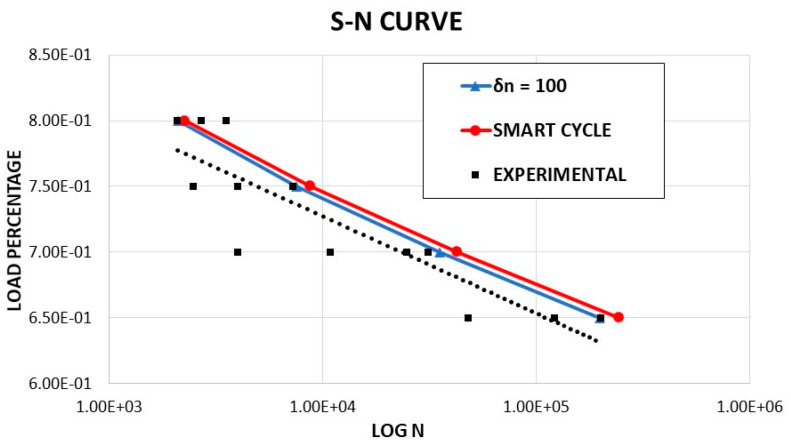
Off-axis tensile specimen: S–N curves.

Fatigue simulations were performed with *R* = 0.1 and maximum load corresponding to 80, 75, 70, and 65% of the maximum static tensile load. The maximum number of cycles was fixed to NTOT = 1 × 10^6^ with an iteration increment of *δn* = 100 cycles for the standard fatigue simulation. The obtained numerical results, both with standard fatigue simulation and with the application of the Smart Cycle strategy, were compared to experimental data from the literature [35]. In Figure 7, the S–N curves numerically evaluated (with standard and Smart Cycle strategy) were compared to the experimental data by Shokrieh and Lessard in [35] (the black dotted line represents the fitting of the experimental points on the graphs). Good agreement was found in terms of number of cycles to failure for all the analyzed applied load levels. In particular, both methods provided excellent agreement at the maximum load case, while, for the other loads, a slight overestimation up to 20% was found between experimental and numerical data. This demonstrates the robustness of the implemented procedure (whose basic empirical model has been extensively validated in the literature) and highlights the fairness of the proposed Smart Cycle strategy and the assumptions on which it is based. Indeed, from Figure 7, it can be observed that the Smart Cycle strategy is able to mimic the physical structural behavior of the specimen under fatigue by providing a response very similar to the standard and computationally heavy fatigue procedure with constant cycle increments.

**Figure 8:**
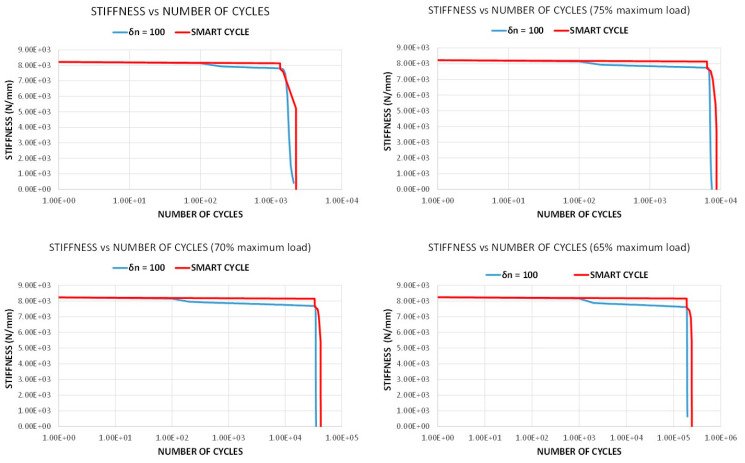
Off-axis tensile specimen: stiffness vs. number of cycles.

Figure 8 compares the material stiffness degradation due to fatigue loads as a function of the number of cycles, obtained with the Smart Cycle strategy and with the standard procedure with constant *δn* = 100 cycle increments, for all the investigated load levels. According to this figure, the stiffness curves obtained with the standard fatigue simulation show a decreasing trend due to the gradual degradation of the mechanical material properties as the number of cycles increases before a consistent decrease of stiffness due to the sudden degradation. On the other hand, only a sudden drop in the stiffness can be observed with the application of the Smart Cycle strategy, which, as already remarked, is based on the hypothesis of negligible effects of the stress redistribution due to the gradual degradation of material properties in the cycle prediction phase. Actually, Figure 8 shows that an overestimation from 8 to 18% in the prevision of the number of cycles to failure can be obtained with the application of the Smart Cycle strategy, which is within the experimental data scatter. This discrepancy is associated with the neglection of the gradual degradation of material properties in the prediction phase of the Smart Cycle strategy, which causes, in case of comparison with a small constant-cycle increment strategy, a delay in the sudden degradation and, consequently, in final failure.

**Figure 9:**
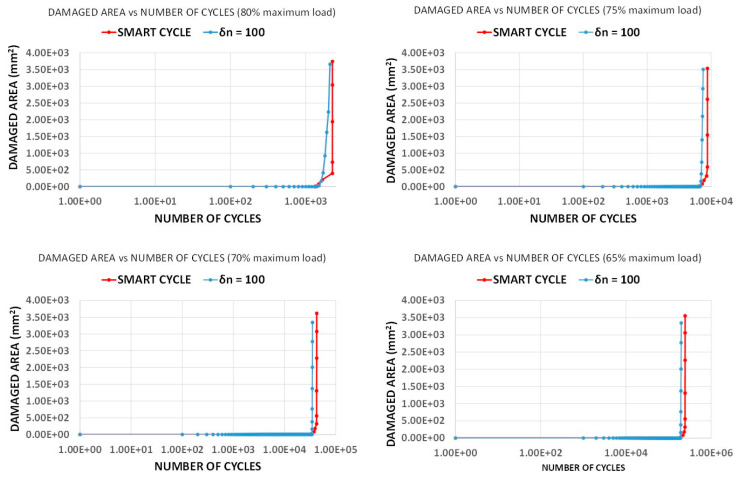
Off-axis tensile specimen: damaged area vs. number of cycles.

**Figure 10:**
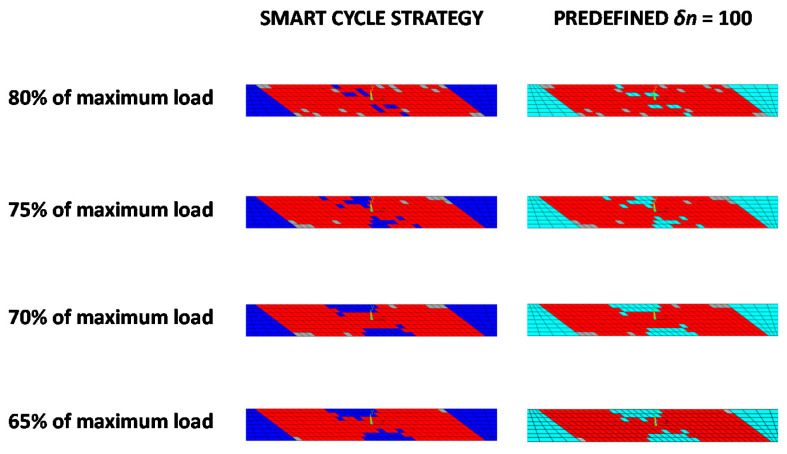
Off-axis tensile specimen: comparison of the final damage state obtained with the Smart Cycle strategy and with constant *δn* = 100 cycle intervals.

The comparisons of the two considered strategies in terms of damaged area evolution as a function of the number of cycles are presented in Figure 9 for all the analyzed load levels. The extent of damaged area at failure is almost identical to the two analyzed numerical strategies. This is also confirmed in Figure 10, where the damage propagation status at final failure, obtained with the two investigated strategies, is shown to be exactly the same (red elements represent the matrix failure and gray elements represent the fiber failure; an element is reported as broken when at least one lamina is broken according to the specific failure mode).

**Figure 11:**
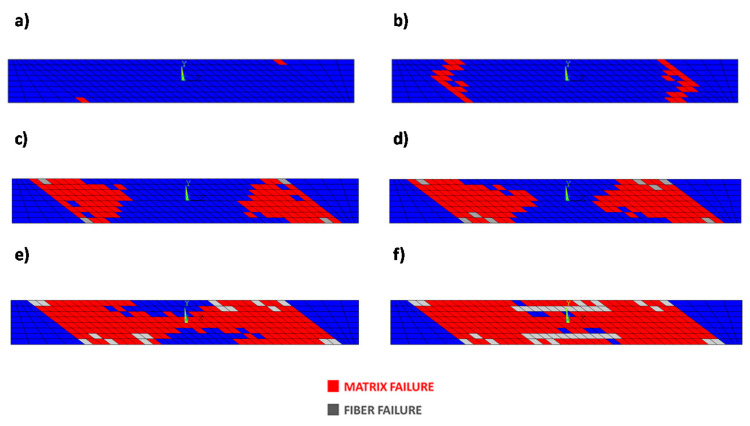
Off-axis tensile specimen: damage status at cycles number (**a**) 1343, (**b**) 1488, (**c**) 1658 (**d**) 2255, (**e**) 2257, and (**f**) 2259 at 80% of the static load using the Smart Cycle strategy.

The damage evolution, as the number of cycles increased (matrix damage initiation at fatigue cycle 1343, intermediate growth at fatigue cycle 1488, fiber damage initiation at fatigue cycle 1658, intermediate growth at fatigue cycles 2255 and 2257, and final damage state at fatigue cycle 2259), for the configuration with 80% of the maximum static load, is shown in Figure 11.

**Figure 12:**
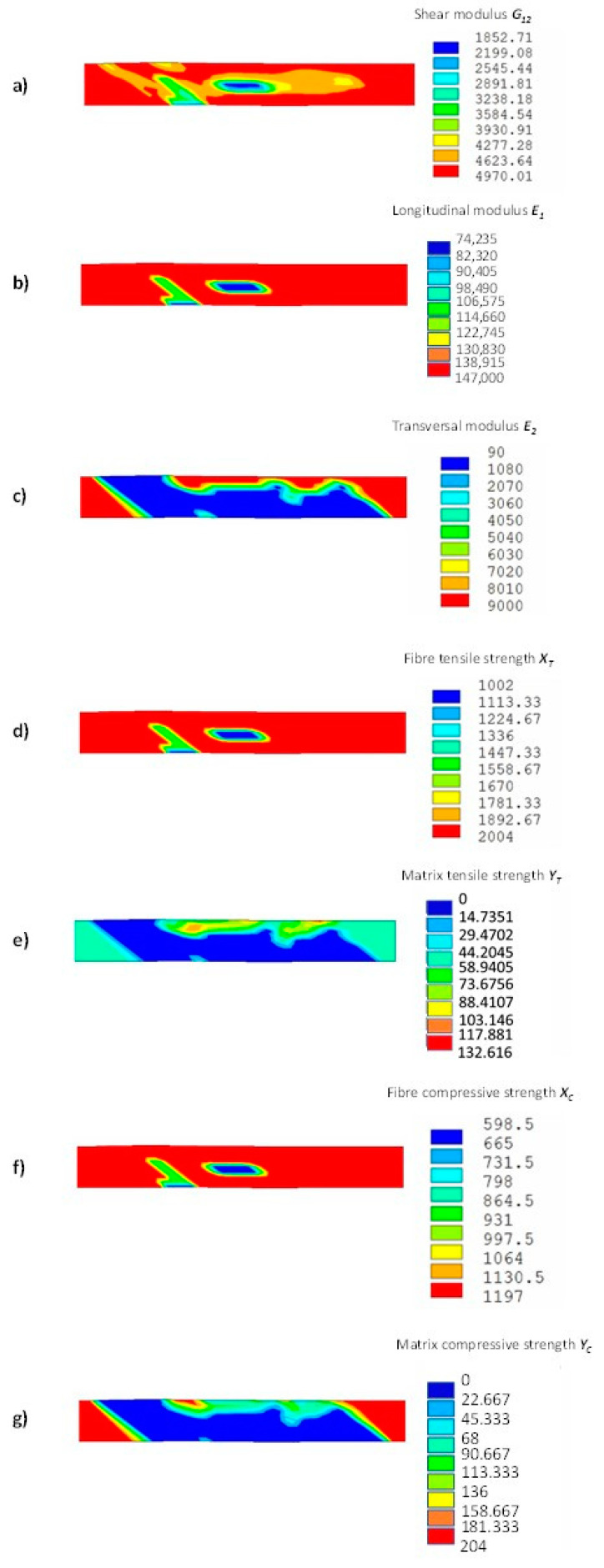
Off-axis tensile specimen with gradual degradation of material properties using the Smart Cycle strategy: (**a**) shear modulus (MPa), (**b**) longitudinal modulus (MPa), (**c**) transversal modulus (MPa), (**d**) longitudinal tensile strength (MPa), (**e**) transversal tensile strength (MPa), (**f**) longitudinal compressive strength (MPa), and (**g**) transversal compressive strength (MPa).

In Figure 12, the distributions of the material properties (stiffness and strength) at the end of the analysis, taking into account the gradual fatigue degradation, obtained with the Smart Cycle routine, are shown. It is clear that, even if the Smart Cycle routine neglects the gradual degradation of material properties when searching for the next fatigue cycle to be simulated, the reduction in material properties with cycles is taken into account when the fatigue stress analysis is performed.

**Figure 13:**
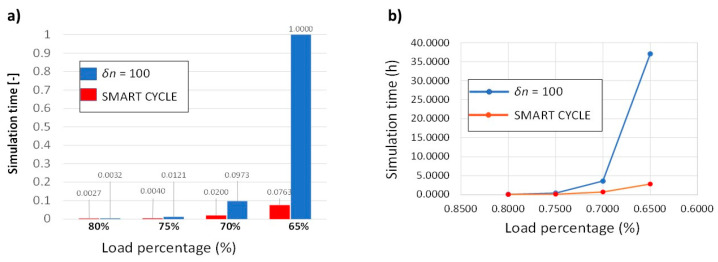
Off-axis tensile specimen: (**a**) simulation time bar chart, (**b**) simulation time vs load percentage.

**Figure 14:**
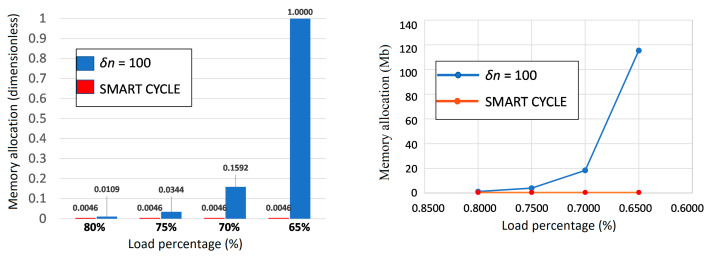
Off-axis tensile specimen: (**a**) hard disk memory allocation bar chart; (**b**) hard disk memory allocation vs. load percentage.

With the aim of assessing the advantage of the Smart Cycle strategy, comparisons in terms of simulation time and memory allocation are presented, respectively, in Figure 13 and Figure 14. Simulation time and memory allocation were normalized with respect to the maximum values obtained for the simulation at 65% of the static load.

From Figure 13 and Figure 14, the advantages of the Smart Cycle strategy with respect to the standard procedure with constant cycle intervals can be appreciated. Actually, even with the approximation introduced by neglecting the gradual degradation in the prediction phase, the Smart Cycle strategy is able to lower the simulation times tenfold and memory allocation 20-fold with respect to the standard procedure with constant cycle intervals. Indeed, relative differences up to −92% in the simulation time and 95% in the memory allocation are achieved with the proposed strategy. The advantages of the Smart Cycle strategy, in terms of simulation time and memory allocations, increase when performing fatigue simulations at lower max loads.

### 4.2. Cross-Ply Open-Hole Tensile Specimen

The presence of cutouts within composite structures is needed for running electrical cables and fuels or just for lowering the weight of the structure. However, holes develop a high stress concentration, which can cause premature collapse of the structure. Hence, the study of the fatigue behavior of components characterized by the presence of cutouts becomes mandatory for structural design.

An AS4/3501–6 carbon/epoxy laminate, with a central circular hole and (0_2_,90_2_)_s_ layup, subjected to tensile fatigue loading conditions, is analyzed in this subsection, and the numerical results in terms of elastic strain and fatigue damage progression, obtained with the Smart Cycle strategy and with the procedure using standard constant cycle intervals, are compared to experimental results by Krishnan et al. in [40] to further validate the Smart Cycle Strategy.

**Figure 15:**
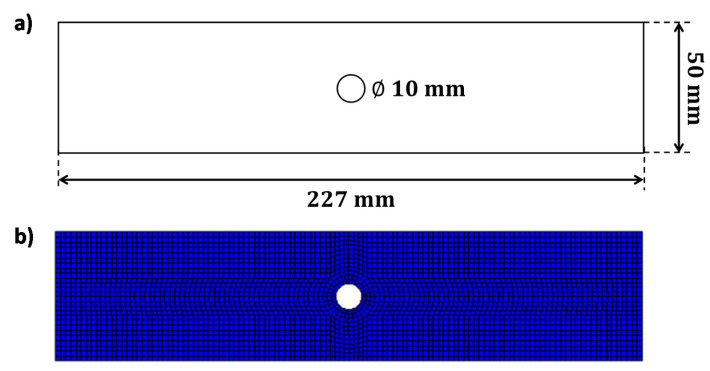
Cross-ply open-hole specimen: (**a**) geometrical model; (**b**) FEM model.

The geometry of the investigated open-hole tension specimen is shown in Figure 15a. The laminate was discretized by using four-node SHELL181 ANSYS layered elements with reduced integration (see Figure 15b). The fatigue test at P_max_ = 25 kN, corresponding to the 56.8% of the static tensile strength, and *R* = 0, was considered.

**Figure 16:**
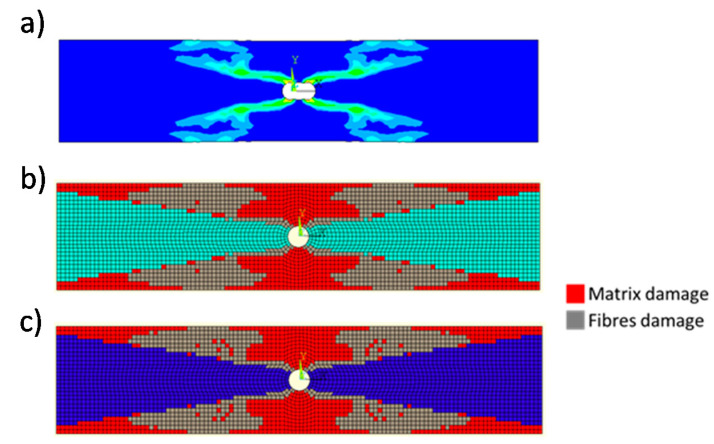
Cross-ply open-hole specimen: (**a**) numerical maximum principal strain; (**b**) final damage status obtained by considering constant cycle increments *δn* = 100; (**c**) final damage status obtained by using the Smart Cycle strategy.

A photo of the numerically predicted maximum first principal strain contour plot at the numerical cycles to failure *N_f_* = 30,784 cycles, obtained with the Smart Cycle strategy, is shown in Figure 16a. The numerical final damage status, obtained with the procedure based on standard constant cycle increments (*δn* = 100 cycles) and with the application of the Smart Cycle strategy, are respectively presented in Figure 16b (*N_f_* = 33,500) and Figure 16c (*N_f_* = 30,784).

From Figure 16, the shear out damage pattern predicted by the implemented numerical models is in good agreement with the experimentally observed damage pattern, which can be found in Figure 10a of [40] by Krishnan et al. The same can be said for numerical cycles to failure (*N_f_* = 30,784 with the Smart Cycle strategy and *N_f_* = 33,500 with the constant cycle increments) and experimental cycles to failure (*N_f_* = 35,000).

**Figure 17:**
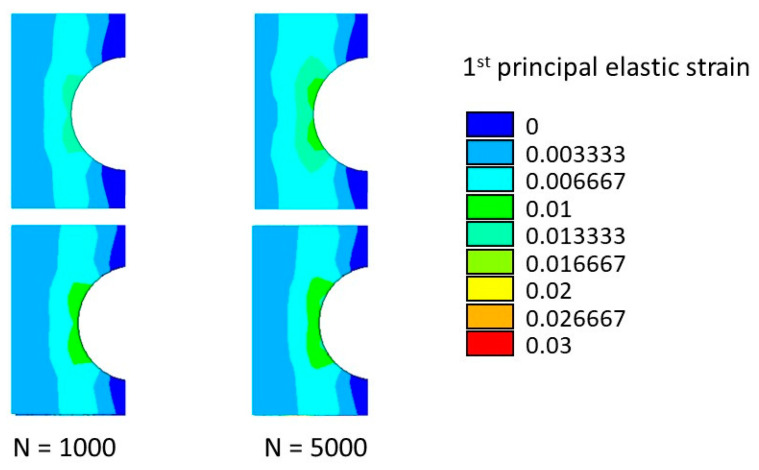
Cross-ply open-hole specimen: maximum first principal strain at 1000 and 5000 cycles.

**Figure 18:**
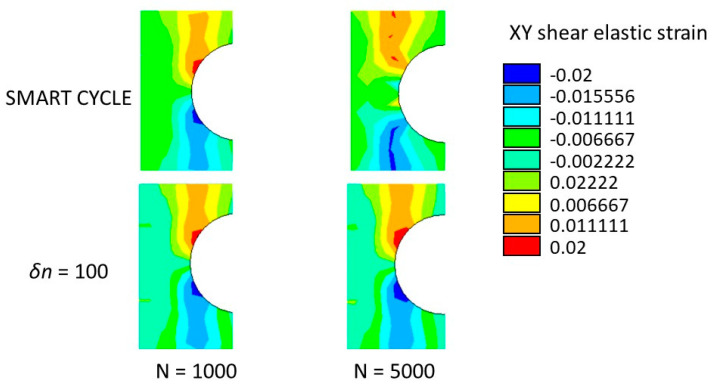
Cross-ply open-hole specimen: *XY* shear strain at 1000 and 5000 cycles.

The numerically predicted strains at *N* = 1000 and *N* = 5000 are shown in Figure 17 (first principal elastic strain) and Figure 18 (*XY* shear elastic strain).

According to Figure 17 and Figure 18, an acceptable agreement was found between the numerical results and the experimental data presented in Figure A2 and Figure 13 by Krishnan et al. in [40] by means of the digital image correlation during the tensile–tensile fatigue experimental test.

**Figure 19:**
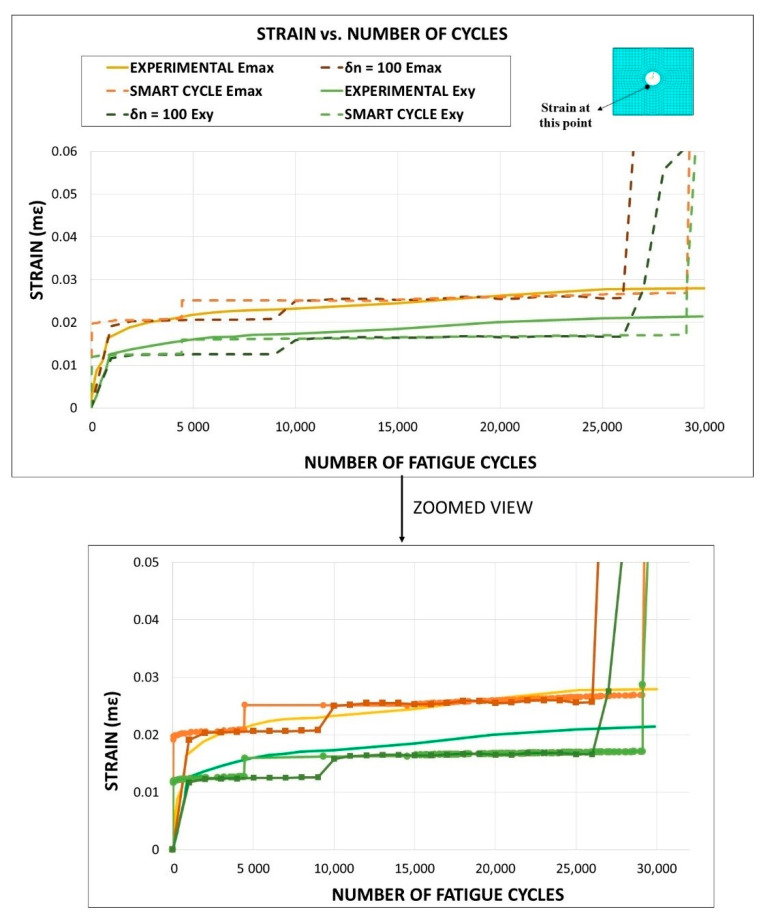
Cross-ply open-hole specimen: strain vs. number of cycles.

In Figure 19, the first principal and the tangential shear strain evolution, near the hole normally to the load application direction, is compared to the Digital Image Correlation (DIC) measured strain evolution. A good correlation between predicted and measured strains, for both strain components, was found up to the numerical fatigue failure phase (between *N_f_* = 25,000 and *N_f_* = 30,784/*N_f_* = 33,500), demonstrating the ability of the implemented numerical models (particularly the Smart Cycle model) to correctly predict the strain evolution under fatigue loading conditions.

**Figure 20:**
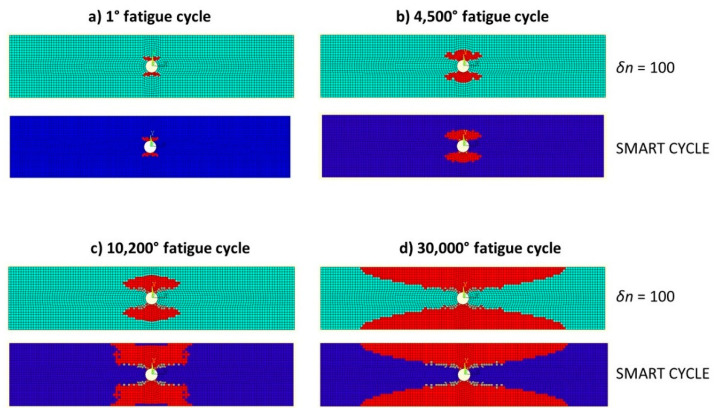
Cross-ply open-hole specimen: damage status at cycle numbers (**a**) 1, (**b**) 4500, (**c**) 10,200, and (**d**) 30,000.

**Figure 21:**
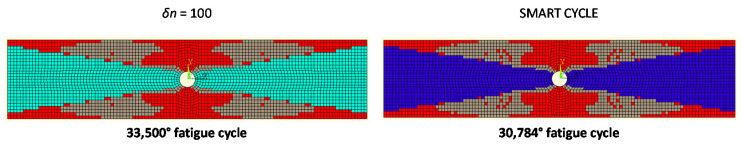
Cross-ply open-hole specimen: comparison between the final damage state obtained with the Smart Cycle strategy and *δn* = 100 cycles.

The damage propagation patterns, obtained with the Smart Cycle strategy and the procedure based on standard constant cycle increments (with *δn* = 100 cycles), are compared in Figure 20, where the red elements represent the matrix failure and the gray elements represent the fiber failure. Again, according to Figure 20, an element is reported as broken when at least one lamina is broken following a specific failure mode. Four different damaged maps are compared, corresponding to four different fatigue cycle numbers (*N* = 1, *N* = 4500, *N* = 10,200, and *N* = 30,000). The final damage states predicted with the two numerical strategies are compared in Figure 21. An 8.8% difference in predicted number of cycles to failure was found between the results obtained using the Smart Cycle procedure and using the procedure based on standard constant cycle increments. This difference is similar to that found when analyzing the previous test case. However, in this case (characterized by large constant cycle increments), it can be stated that the neglection of the gradual degradation in the prediction phase of the Smart Cycle procedure induced a slight underestimation of the number of cycles to failure.

**Figure 22:**
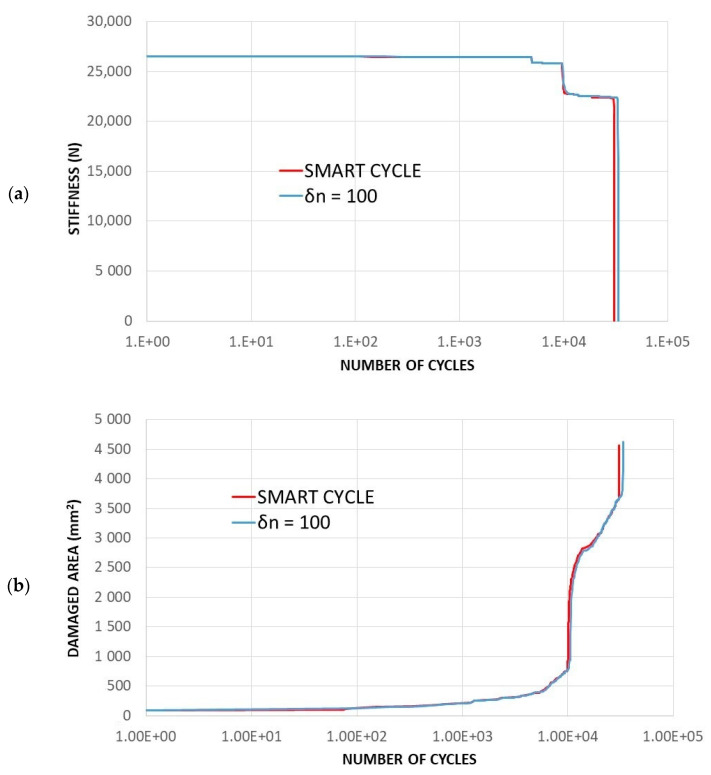
Cross-ply open-hole specimen—comparison between Smart Cycle and standard procedure: (**a**) numerically predicted stiffness vs. number of cycles; (**b**) numerically predicted damaged area vs. number of cycles.

In Figure 22, the stiffness degradation and the damaged area trends as a function of the number of cycles, for the two investigated numerical procedures, are presented, where very slight differences can be observed. This figure confirms that the Smart Cycle procedure slightly underestimates the number of cycles to failure with respect to the procedure based on large constant cycle increments.

**Figure 23:**
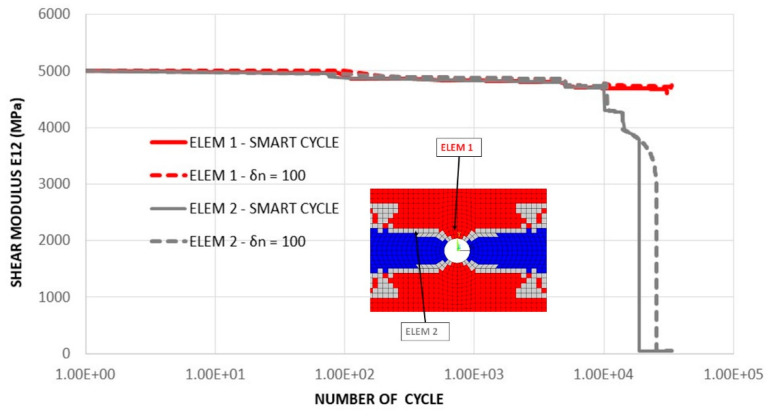
Cross-ply open-hole specimen—comparison between Smart Cycle and standard procedure: shear modulus *E*_12_ vs. number of cycles.

In order to highlight the differences in material property degradation at different specimen locations during fatigue, in Figure 23, the degradation of the shear modulus *E*_12_ as a function of the fatigue cycles in two finite elements is shown. The first element did not experience fiber failure; hence, the shear modulus decreased by up to 5.3% during the analysis. The second element underwent fiber failure at fatigue cycle 18,824 according to the Smart Cycle procedure and 25,600 according to the procedure based on standard constant cycle increments (with *δn* = 100).

**Figure 24:**
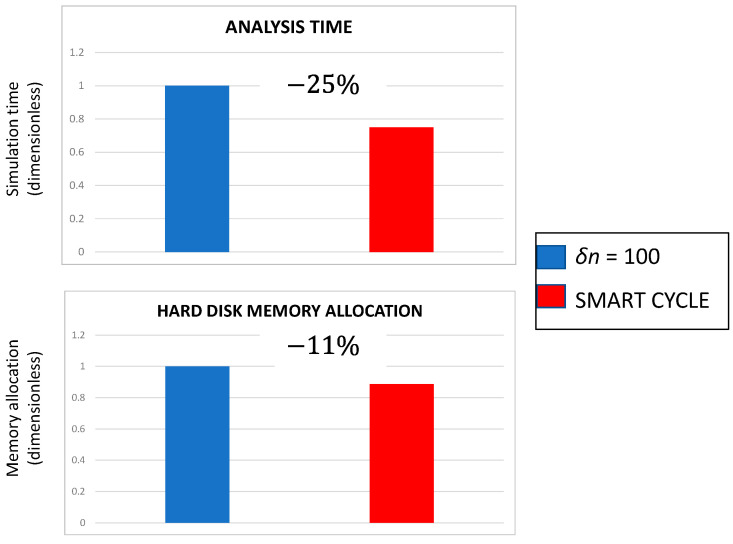
Cross-ply open-hole specimen—comparison between Smart Cycle and standard procedure: simulation time and memory allocation.

The comparison in terms of simulation time and hard disk memory allocation, reported in Figure 24, gives an idea of the advantages gained with the Smart Cycle strategy if compared to the procedure based on standard constant cycle increments (with *δn* = 100). In this case, when compared with the procedure based on large constant cycle increments, the Smart Cycle strategy still showed advantages in terms of simulation time and hard disk memory allocation. The performances of the Smart Cycle strategy can be increased, as mentioned in the previous subsection, by tailoring the cycle intervals at which the prediction of damage cycles should be performed.

## 5. Conclusions

This work dealt with the fatigue response of composite materials, and it was based on Shokrieh and Lessard’s residual strength material property degradation model. A finite element methodology was implemented in the commercial software ANSYS MECHANICAL^®^ (v18.0, 2018, Ansys, Inc., Canonsburg, PA, USA) through the Ansys Parametrical Design Language (APDL) to obtain a robust and easy-to-use numerical procedure for the preliminary assessment of the fatigue life of composite structural components. Shokrieh and Lessard’s model was enhanced with a novel cycle jump strategy, called the Smart Cycle strategy, to estimate the cycles where fatigue failure criteria are presumably verified and to reduce the computational costs in terms of time and memory allocation. The Smart Cycle strategy’s principal hypothesis is that the stress redistribution, due to the fatigue-induced gradual degradation of the material properties, is negligible until sudden fiber and/or matrix damage is verified at the element/lamina level due to cyclic loading. Hence, the number of fatigue stress analyses is considerably reduced if compared with the standard fatigue literature models, where a predefined cycle increment is considered, without loss of result accuracy. This is the main added value of the proposed fatigue damage approach.

A preliminary validation of the developed procedure was performed by comparing numerical results from the Smart Cycle strategy to standard numerical models based on constant cycle increments and literature experimental data. First, the tensile–tensile fatigue behavior of an off-axis specimen, considering an applied load fraction (80, 75, 70, and 65%) of the static maximum tensile load, was simulated. The numerically predicted S–N curve, compared to the experimental data by Shokrieh and Lessard in [35], showed good agreement, in terms of number of cycles to failure, particularly for 80% of the maximum load. A slight overestimation (up to 20%) of the Smart Cycle strategy prediction, due to the neglection of gradual fatigue degradation in the prediction phase of the module, was noted when compared to procedures with small constant cycle increments.

Then, a cross-ply sample with circular cutout, subjected to constant-amplitude tensile fatigue, considering a load of 56.8% of the static tensile strength, was investigated. A good correlation was found when comparing experimental and numerical results from the standard procedure with constant cycle increments to the Smart Cycle strategy numerical results. Indeed, acceptable agreement was found in terms of strains by comparing the literature measurements obtained by digital image correlation (DIC) and the numerically predicted ones, demonstrating the capability of the implemented approximated numerical strategy to correctly predict the physical damage pattern. For this second test case, an underestimation of the number of cycles to failure of about 8% was found with respect to the procedure based on large constant cycle increments.

For the two considered test cases, the Smart Cycle strategy was found able to decrease the simulation time and the memory allocation size up to tenfold with respect to the procedures based on large and small standard constant cycle increments, providing affordable results within the range of 8–18% in terms of number of cycles to failure. Hence, the developed procedure enables obtaining a preliminary assessment of the fatigue behavior of composite materials with the same accuracy of literature standards and with excellent reduction in terms of computational costs. This may be useful in the preliminary design phase of composite material structure.

## Figures and Tables

**Table 1:** Hashin fatigue failure criteria.

Failure Modes	Equations	Parameters
Tensile fiber failure	${\left(\frac{\sigma_{11}}{X_{T}\left(n,\;\sigma ,\;k\right)}\right)}^{2}+{\left(\frac{\sigma_{12}}{S\left(n,\;\sigma ,\;k\right)}\right)}^{2}=1$	$X_{T}\left(n,\sigma ,k\right)$ fiber tensile fatigue strength S(n,σ,k) shear fatigue strength
Compressive fiber failure	${\left(\frac{\sigma_{11}}{X_{C}\left(n,\;\sigma ,\;k\right)}\right)}^{2}=1$	$X_{C}\left(n,\sigma ,k\right)$ fiber compressive fatigue strength
Tensile matrix failure	${\left(\frac{\sigma_{22}}{Y_{T}\left(n,\;\sigma ,\;k\right)}\right)}^{2}+{\left(\frac{\sigma_{12}}{S\left(n,\;\sigma ,\;k\right)}\right)}^{2}=1$	$Y_{T}\left(n,\sigma ,k\right)$ matrix tensile fatigue strength S(n,σ,k) shear fatigue strength
Compressive matrix failure	${\left(\frac{\sigma_{22}}{Y_{C}\left(n,\;\sigma ,\;k\right)}\right)}^{2}+{\left(\frac{\sigma_{12}}{S\left(n,\;\sigma ,\;k\right)}\right)}^{2}=1$	$Y_{C}\left(n,\sigma ,k\right)$ matrix compressive fatigue strength

**Table 2:** Material properties.

**Property**	**Value**	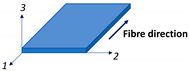
$E_{11}$	147,000 MPa	
$E_{22}=E_{33}$	9000 MPa	
$G_{12}=G_{13}$	5000 MPa	
$G_{23}$	3000 MPa	
$\nu_{12}=\nu_{13}$	0.3 (-)	
$\nu_{23}$	0.42 (-)	
$X_{T}$	2004 MPa	
$X_{C}$	1197 MPa	
$Y_{T}=Z_{T}$	53 MPa	
$Y_{C}=Z_{C}$	204 MPa	
$S_{XY}=S_{XZ}$	137 MPa	
$S_{YZ}$	42 MPa	

## Data Availability

The raw/processed data required to reproduce these findings are available throughout the manuscript.
